# Haemostatic alterations in a group of canine cancer patients are associated with cancer type and disease progression

**DOI:** 10.1186/1751-0147-54-3

**Published:** 2012-01-26

**Authors:** Eva B Andreasen, Mikael Tranholm, Bo Wiinberg, Bo Markussen, Annemarie T Kristensen

**Affiliations:** 1 Department of Small Animal Clinical Sciences, Faculty of Life Sciences, University of Copenhagen, Denmark; 2 Haemostasis Pharmacology, Novo Nordisk A/S, Denmark; 3 Department of Basic Sciences and Environment, Biostatistics and Mathematics, Faculty of Life Sciences, University of Copenhagen, Denmark

**Keywords:** Haemostasis, Cancer, Dogs, Thromboelastography, Metastases

## Abstract

**Background:**

Haemostatic alterations are commonly detected in human and canine cancer patients. Previous studies have described haemostatic dysfunction in canine patients with haemangiosarcomas and carcinomas, and haemostasis has been assessed in dogs with various malignant and benign neoplasias. Few studies have addressed the effect of cancer type and progression of disease on the presence of haemostatic alterations in canine patients. The objective of the present study was to evaluate haemostatic variables of coagulation and fibrinolysis in a group of canine cancer patients, and to compare haemostatic changes to the cancer type and progression of disease.

**Methods:**

The study population consisted of 71 dogs with malignant neoplasia presented to the University Hospital for Companion Animals, Faculty of Life Sciences, University of Copenhagen, Denmark. The study was designed as a prospective observational study evaluating the haemostatic function in canine cancer patients stratified according to type of cancer disease and disease progression. The coagulation response was evaluated by thromboelastrography (TEG), platelet count, activated partial thromboplastin time (aPTT), prothombin time (PT), fibrinogen and antithrombin (AT); and fibrinolysis by d-dimer and plasminogen.

**Results:**

Hypercoagulability was the most common haemostatic dysfunction found. Non mammary carcinomas had increased clot strength (TEG G), aPTT and fibrinogen compared to the other groups. When stratifying the patients according to disease progression dogs with distant metastatic disease exhibited significantly increased fibrinogen, and d-dimer compared to dogs with local invasive and local non-invasive cancers.

**Conclusion:**

Hypercoagulability was confirmed as the most common haemostatic abnormality in canine cancer patients and haemostatic dysfunction in canine cancer patients was found related to the cancer type and progression of disease. Increase in TEG G, aPTT and fibrinogen were observed in non-mammary carcinomas and were speculated to overall represent a proinflammatory response associated with the disease. Dogs with distant metastatic disease exhibited increased fibrinogen and d-dimer. Future studies are needed to elucidate the clinical importance of these results.

## Background

Haemostatic dysfunction is a common finding in human cancer patients who often present with clinical signs of venous thromboembolism (VTE), pulmonary thromboembolism (PTE) or low grade disseminated intravascular coagulation (DIC) [1,2]. On average 1.5% of human cancer patients develop clinical signs of VTE or PTE, with metastatic disease at the time of diagnosis being the strongest predictor of VTE. In addition, the incidence of VTE is found to vary with cancer type [3,4]. Subclinical findings may include abnormal haemostatic parameters, i.e. elevation of fibrinogen, fibrin degradation products (FDP) and thrombocytosis [5]. Elevated fibrinopeptide A and d-dimer levels have been found associated with persistent disease activity and tumor burden [6,7].

In dogs, cancer has been associated with PTE, though the condition is rarely diagnosed ante-mortem [8,9]. As in humans DIC has been reported in canine cancer patients with metastasizing mammary carcinoma [10], patients with acute lymphoblastic leukemia [11] and in dogs with haemangiosarcomas [12]. Subclinical haemostatic alterations associated with cancer in canine patients appear to be common. Madewell *et al. *[13] found abnormal coagulation parameters in 83% of tumor bearing dogs with advanced disease including decreased and increased fibrinogen level, thrombocytopenia, prolongation of activated partial thromboplastin time (aPTT) and increased FDP. Another, more recent study, found abnormalities in 67% of 60 female dogs with mammary carcinoma, with the frequency and likelihood increasing for stage III and IV disease [14]. In a recent study hypercoagulability, thrombocytosis and increased fibrinogen was the most common finding in a population of 32 canine carcinoma patients compared to 19 healthy controls, though no association between haemostatic changes and presence of metastatic disease could be made [15].

Thromboleastography (TEG) is a method to evaluate whole blood coagulability. Only few studies have been published in human cancer patients, but in other clinical settings, hypercoagulability as measured by TEG, has been found to correlate with thrombotic complications [16,17]. A study in 78 human cancer patients demonstrate TEG hypercoagulability in patients with solid tumors but was unable to correlate TEG parameters to sub-groups of different cancer types or stages of disease according to the "tumor-node-metastasis" staging system [18].

In a previous study, Kristensen *et al. *[19] found haemostatic dysfunction in 57% of dogs with neoplasia. TEG analysis demonstrated these patients to be primarily hypercoagulable as in human cancer patients. Furthermore, patients with malignant tumors had a significantly higher degree of haemostatic dysfunctions compared to patients with benign tumors. In addition, albeit few, patients with hypocoagulable TEG G all had metastatic disease. Due to incomplete staging procedures and a low number of patients for each tumor type, the relationship between haemostatic dysfunction and cancer tissue type or progression of cancer disease was not examined.

The objective of the present study was to evaluate haemostatic variables of coagulation and fibrinolysis in a group of canine cancer patients and to compare haemostatic changes to cancer type and progression of disease. We hypothesized that haemostatic dysfunction in canine cancer is related to the type and progression of disease and whether the disease is local, regional or if distant metastases are present.

## Materials and methods

This study was approved by the Small Animal Ethics and Administrative Committee at the Department of Small Animal Clinical Sciences, Faculty of Life Sciences, University of Copenhagen Denmark.

### Study population

The study was performed as a prospective observational study, during a three year period from 2007 to 2010 at the University Hospital for Companion Animals, Department of Small Animal Clinical Sciences, Faculty of Life Sciences, University of Copenhagen. The study included 71 dogs and the mean age of the patients was 8 years and 3 month, (range 1.5 - 14.6 years). The study population included 52 females of which 11 were spayed and 19 males of which 5 were castrated.

The clinical and paraclinical evaluation of the patients included physical examination, evaluation of palpable draining lymph nodes by fine needle aspiration cytology, hematology, complete biochemistry profile, coagulation and fibrinolysis parameters (aPTT, PT, platelet count, fibrinogen, antithrombin, d-dimer, plasminogen) and TEG. Staging procedures as recommended by Withrow and Vail [20] were attempted for each cancer type and in addition to the above evaluation consisted of radiographs or CT scan of the thorax and/or ultrasound examination of the abdomen. Diagnosis was obtained by histopathology for all tumors except for lymphoma and mastocytoma where a diagnosis based on cytology was accepted. Patients were excluded from the study if they were found to have non-malignant tumors or if they were diagnosed with concurrent illness unrelated to the cancer diagnosis that was expected to influence the data. Patients treated with NSAIDs or steroids within the last 14 days before they were admitted to the Hospital were excluded as well. None of the patients had received chemotherapy prior to inclusion.

To evaluate the influence of cancer type on the haemostatic parameters, the patients were grouped according to their cancer type as having either mammary gland carcinoma (n = 23, carcinoma - mammary), carcinomas located at other sites than the mammary gland (n = 7; carcinoma - other site), osteosarcoma (n = 6), soft tissue sarcomas (n = 13), mastocytoma (n = 12), or lymphoma (n = 10).

To evaluate the influence of progression of disease on the haemostatic parameters, patients were grouped as having presence of local disease without invasion ("local non-invasive"; n = 3), local disease with invasion ("local invasive"; n = 37), regional metastasis (n = 5) or distant metastatic disease (n = 10). Patients were defined to have local disease without invasion if the tumor by histopathology had no signs of invasive growth, and patients were grouped as having local disease with invasion if the tumor by histopathology had signs of invasive growth. Patients were grouped as having regional disease if the fine needle aspiration cytology from draining lymph nodes were found to contain malignant cells, and to have distant metastatic disease if cytology and/or imaging showed presence of distant metastasis. Patients with lymphoma or a diagnosis by cytology (six dogs with mastocytoma) were not included in this part of the study.

The distribution of i) patients according to cancer type, diagnosis by histopathology and progression of disease, ii) conducted staging procedures and iii) site of primary and distant sites of cancer for the patients with distant metastases are shown in details in additional files 1, 2 and 3: [see Additional file 1, 2 and 3].

### Blood sampling

Blood was collected during the initial admission to the Hospital. Whole blood was collected by careful venipuncture using minimal stasis and a 21-G butterfly needle. A total maximum blood volume of 2% of the body weight was collected from each patient. Blood for haemostatic parameters was collected into citrated BD Vacutainers and was obtained at the same time as blood for routine haematology and biochemistry. After collection, the citrate tubes were carefully inverted 5 times to ensure mixing of 3.2% trisodium citrate and blood in a 1:9 ratio. Whole blood for TEG analysis was stored at room temperature (around 20°C) until the measurement was performed 30 minutes after sampling. Blood was centrifuged at 4,000 × g for 120 seconds, and aPTT, PT, fibrinogen and d-dimer analyses were performed within 30 minutes of blood sampling. In addition plasma was aliquoted and stored at -80°C.

### Hematology and biochemistry

A complete blood count (CBC) consisting of a white blood cell count, concentration of neutrophils, lymphocytes, monocytes, eosinophils, basophils, erythrocytes, evaluation of haemoglobin and haematocrit were evaluated on all patients using EDTA stabilized whole blood and an automated haematology analyzer (Advia 120, Siemens Healthcare Diagnostics). Biochemical profiles were measured on serum samples using an automated biochemistry analyzer (Advia 1800 Chemistry System, Siemens Healthcare Diagnostics).

CBC and biochemistry were performed at the Central Laboratory, Department of Small Animal Clinical Sciences.

### Thromboelastography

The TEG measurements were conducted as previously described [21]. In brief, recombinant human TF (Innovin, Dade Behring) was used as activator at a final concentration of 1:50.000. The TEG analysis was initiated 30 minutes after the blood was collected. Re-calcification with 20 μL 280 mmolar CaCl_2 _was added to each pre-warmed cup. Finally 20 μL prediluted TF was mixed with 320 μL canine citrated whole blood and the premix added to the cup, giving a total volume of 360 μL/cup. The TEG analysis was run for 120 minutes. The TEG parameters R, K and Angle (Clot kinetics), MA and G (Clot strength), Ly30 and Ly60 (fibrinolysis) were evaluated for all patients as previously reported [19,22]. G was calculated from MA with G = 5,000 × MA/(100 - MA) and is a measure of the overall coagulant state as normo-, hyper-, or hypo-coagulant. Patients were considered to be normocoagulable when the TEG G value was within the normal range of 3.2-7.2 × 10^3 ^dyn/cm^2^. A TEG G value below the normal range was considered as hypocoagulable and values above defined as hypercoagulable [23].

### Coagulation tests

All haemostatic tests were performed at the Central Laboratory employing commercial reagents in a validated setup with an automated coagulometer (ACLTop 500, Instrumentation Laboratory) as previously described [24]: aPTT (SynthAFax, Instrumentation Laboratory); PT (RecombiPlasTin 2G, Instrumentation Laboratory); d-dimer (D-Dimer Single Tests, NycoCard READER II, Medinor A/S); fibrinogen (RecombiPlasTin 2G, Instrumentation Laboratory); AT (Liquid Antithrombin; Instrumentation Laboratory) and plasminogen (Plasminogen, Instrumentation Laboratory). A pooled sample of plasma from ten clinical healthy dogs was analyzed together with the patient samples and used as internal control. Platelet count was measured using an automated haematology analyzer (Advia 120) using EDTA stabilized whole blood. Normal values for the clot assay of aPTT, PT, fibrinogen and d-dimer were based on internal validation and normal values of 30 clinical healthy dogs. Due to technical failures and lost samples, aPTT was missing for 10 dogs, PT was missing in two dogs, fibrinogen was missing in two dogs, AT missing in 16 dogs and plasminogen missing in 15 dogs.

### Statistical analysis

Data was tested for normal distribution using Shapiro-Wilk test for normality. Since values were not normally distributed, the statistical analysis was carried out as non-parametric. Kruskal Wallis test was used for comparison of numerical values for multiple groups. When there was overall statistical significance, a Dunn's Post Test for multiple comparisons was used to compare the individual groups.

Statistical calculations were performed using the software program SAS 9.1 edition (SAS Institute Inc) and GraphPad Prism 5 (GrapfPad Software). *P *< 0.05 was chosen as a significant level.

## Results

An abnormal TEG profile was found in 50 of the 71 dogs investigated (70.4%). Forty-seven dogs (66.2%) were hypercoagulable, 3 dogs (4.2%) were hypocoagulable and 21 dogs (29.6%) were normocoagulable. The distribution according to TEG coagulability is displayed in table 1. In the 50 patients with an abnormal TEG hypercoagulability was the most common abnormality (94%). All hypocoagulable patients were found to have systemic cancer disease; one dog had mammary carcinoma (tubulopapillary carcinoma) and distant metastases, one dog had mammary carcinoma (anaplastic carcinoma) and metastases to the draining lymph node and one dog had lymphoma stage V (B-cell origin).

**Table 1:** Distribution of patients based on thromboelastography determined coagulability (G), cancer type and disease progression group

TEG G	N(ct)	CM	COS	OS	STS	M	L	N(dp)	DM	RM	LI	LNI
**Normo** (3.2-7.2)	29.6% (21)	21.7% (5)	0% (0)	33.3% (2)	46.2% (6)	41.7% (5)	30% (3)	29.1% (16)	20% (2)	0% (0)	29.7% (11)	100% (3)

**Hyper** (> 7.2)	66.2% (47)	69.6% (16)	100% (7)	66.7% (4)	53.8% (7)	58.3% (7)	60% (6)	67.3% (37)	70% (7)	80% (4)	70.3% (26)	0% (0)

**Hypo** (< 3.2)	4.2% (3)	8.7% (2)	0% (0)	0% (0)	0% (0)	0% (0)	10% (1)	3.6% (2)	10% (1)	20% (1)	0% (0)	0% (0)

**N**	**71**	**23**	**7**	**6**	**13**	**12**	**10**	**55**	**10**	**5**	**37**	**3**

In percentage is the proportion of patients for the given cancer type or disease progression group.

TEG G = Coagulability measured by TEG G; N(ct) = Percentage and number of patients (in total) evaluated by cancer type; CM = Carcinoma located in the mammary gland; COS = Carcinoma located at other sites than the mammary gland; OS = Osteosarcoma; STS = Soft tissue sarcoma; M = Mastocytoma; L = Lymphoma; N(dp) = Percentage and number of patients (in total) evaluated by disease progression group; DM = Distant metastases; RM = Regional metastases; LI = Local invasive; LNI = Local non-invasive.

### Haemostatic changes according to cancer type

**Figure 1:**
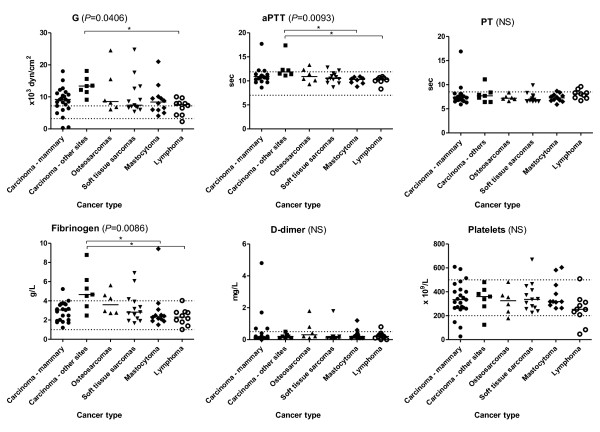
**Thromboelastography G, activated partial thromboplastin time, prothrombin time, fibrinogen, d-dimer and platelet count grouped by cancer type**. The TEG parameter G represents the clot strength. The short solid lines represent the median value for each cancer type. The dotted lines represent the reference interval (G: 3.2-7.2 x10^3 ^dyn/cm^2^; aPTT: 7 - 11.9 sec; PT: < 8.5 sec; fibrinogen: 1-4 g/L; d-dimer: < 0.5 mg/L; platelet count: 200-500 10^9^/L). Overall significance is evaluated by Kruskal Wallis test and indicated by brackets. (NS): non-significant (Kruskal Wallis). Dunn's Post Test was used to test for statistical significance between groups.┌──┐ indicates statistical significance between the groups at each end of the bar. * = Dunn's Post Test: *P *< 0.05.

An overall significant difference was found in TEG clot strength (median G value (*P *= 0.0406; Figure 1) and MA (*P *= 0.0407; data shown in an additional file [see Additional file 4]) between the cancer types. Patients with "Carcinomas at other sites" had a significantly increased TEG G and MA compared to lymphoma patients (*P *< 0.05). No significant differences were found between the different cancer types for the other TEG parameter (R, K, Angle, LY30, and Ly60; data shown in an additional file [seeAdditional file 4]).

An overall significant difference between the cancer types was found for aPTT (*P *= 0.0093; Figure 1) and fibrinogen (*P *= 0.0086; Figure 1). "Carcinomas at other sites" had a significantly increased aPTT (*P *< 0.05) and fibrinogen level (*P *< 0.05) compared to dogs with mastocytoma and lymphoma. No differences between the cancer types were found for PT, d-dimer and platelet count (Figure 1), nor for AT and plasminogen (data shown in an additional file [see Additional file 4]).

### Haemostatic changes according to progression of disease

**Figure 2:**
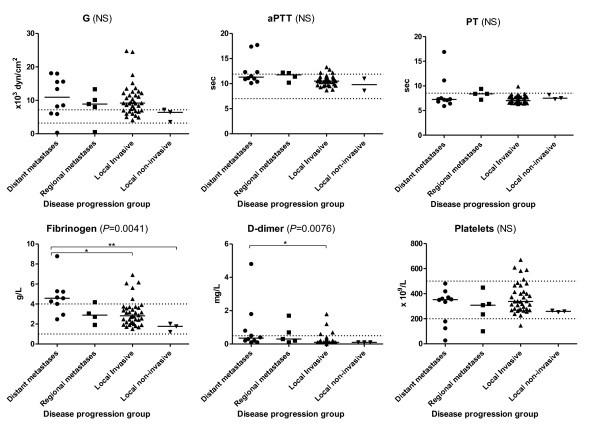
**Thromboelastography G, activated partial thromboplastin time, prothrombin time, fibrinogen, d-dimer and platelet count grouped by progression of disease**. The TEG parameter G represents the clot strength. The short solid lines represent the median value for each disease progression group. The dotted lines represent the reference interval (G: 3.2-7.2 x10^3 ^dyn/cm^2^; aPTT: 7 - 11.9 sec; PT: < 8.5 sec; fibrinogen: 1-4 g/L; d-dimer: < 0.5 mg/L; platelet count: 200-500 10^9^/L). Overall significance is evaluated by Kruskal Wallis test and indicated by brackets. (NS): non-significant (Kruskal Wallis). Dunn's Post Test was used to test for statistical significance between groups. ┌──┐ indicates statistical significance between the groups at each end of the bar. * = Dunn's Post Test: *P *< 0.05. ** = Dunn's Post test: *P *< 0.01.

No statistical significant differences were found between the disease progression groups for TEG G (Figure 2) or any other TEG parameters (data shown in an additional file [see Additional file 5]).

An overall significant difference between the disease progression groups were found for fibrinogen (*P *= 0.0041) and d-dimer (*P *= 0.0076) (Figure 2).

Patients with distant metastases were found to have a significant higher fibrinogen level than patients with local invasive (*P *< 0.05) and local non-invasive cancer (*P *< 0.01), and patients with distant metastases had a significantly elevated d-dimer compared to patients with local invasive disease (*P *< 0.05).

No difference between the disease progression groups were found for aPTT, PT, or platelet count (Figure 2) or for AT and plasminogen (data shown in an additional file: [see Additional file 5]).

## Discussion

In the current study, carcinomas located at other sites than the mammary glands were found to have an increased TEG G value, aPTT and fibrinogen compared to the other evaluated cancer types. In addition patients with distant metastatic disease were found to have elevated levels of fibrinogen and d-dimer compared to patients with local invasive and local non-invasive disease.

All the investigated cancer types in this study exhibited elevated median TEG G values and tended to be hypercoagulable, though the highest TEG G levels were seen in the group of patients with non mammary carcinomas ("carcinomas at other sites"). These patients were also found to have the highest median aPTT and fibrinogen level, even significantly when compared to the groups with mastocytomas and lymphomas.

Fibrinogen levels have previously been shown to positively correlate with TEG G in addition to platelet count [15,22,25]. We speculate, that the fibrinogen level in combination with the slightly elevated median aPTT and the haemostatic changes (elevated TEG G), likely is the net result of a systemic inflammatory reaction seen in these patients, in part mediated through proinflammatory changes in and around the cancer cells [26-28].

The patients with distant metastases were even found to have significantly increased fibrinogen levels compared to patients with local invasive and local non-invasive cancers, which is in accordance with findings made by other authors, who related hyperfibrinogenemia to stage of disease in canine mammary carcinoma patients [14]. Also, increased fibrinogen levels have been associated with progression and extend of cancer disease in human patients [1]. As previously mentioned the elevated fibrinogen seen in patients with metastatic disease indicates that systemic inflammation is associated with progression of the cancer disease.

The patients with distant metastatic disease had a significantly elevated d-dimer level and thereby more pronounced activation of the coagulation and fibrinolytic systems than patients with local invasive disease. Elevated d-dimer levels are found in both human and canine patients with VTE but also in other conditions such as wound healing, DIC and haemorrhagic conditions [29,30]. None of the patients in this study had undergone surgery before blood sampling, but two of the patients with distant metastatic disease were diagnosed with DIC. None of the patients were diagnosed with VTE, though the presence of a thromboembolic event could have been missed. Due to decreased liver clearance, increased d-dimer can be found in patients with cirrhotic liver disease and two of the patients with distant metastasis had elevated liver enzymes. Anyway, it is unlikely that these patients, without clinical signs of liver involvement, were affected by their liver disease to an extend being the sole reason for alterations of their haemostatic and fibrinolytic profiles

Three of the included dogs in this study were found to be hypocoagulable on TEG. Two of the dogs had mammary carcinomas and metastases to either the regional lymph node or distant sites, while the third dog had lymphoma stage V. These findings are in accordance with previous findings by Kristensen *et al. *[19], where hypocoagulable dogs by TEG all had metastatic disease or lymphoma. The limited number of hypocoagulable dogs included in this study is not sufficient to state whether all hypocoagulable cancer patients have systemic cancer disease.

More than 90% of human cancer patients have subclinical haemostatic abnormalities with the majority of patients showing hypercoagulability associated with elevated fibrinogen levels, thrombocytosis, elevated levels of clotting factors V, VIII, IX and X and elevated levels of FDP, in addition to reduced levels of coagulation inhibitors (AT, Protein C and Protein S) and increased levels of plasminogen activator inhibitor-1 [1,5,31]. Similarly, the majority of canine cancer patients have been found to have subclinical haemostatic alterations as were found in this study [13-15,19].

Haemostatic dysfunction and hypercoagulability in cancer patients is complex with a multifactorial aetiology that includes expression of procoagulant molecules such as tissue factor on the surface of malignant cells, release of fibrinolytic peptides, inhibition of endogenous anticoagulation, release of cytokines by cancer cells, and interaction with host cells including endothelial cells and blood leukocytes [28,32]. Increased presence of activated platelets and release of platelet micro vesicles has also been related to hypercoagulability in cancer [33-35]. All these mechanisms additionally support cancer cell growth and metastasis.

In humans, hypercoagulability measured by TEG has been associated with increased risk of thromboembolism especially in post operative patients [16] but has not yet been identified as a predictor of VTE in cancer patients.

In order to verify the current findings, further studies including more patients of each cancer type and disease progression group should be performed. Furthermore the clinical impact of the hypercoagulable state in cancer patients and its possible association with VTE in both humans and dogs should be pursued. It appears that human and canine cancer patients develop similar haemostatic alterations, though thromboembolic complications are seldom recognized in dogs. The similarities of the haemostatic alterations, supports cancer bearing pet dogs as a valuable clinical translational animal model for studying the bidirectional link between haemostasis and cancer in humans.

## Conclusions

Haemostatic alterations were related to disease progression and type of cancer in this population of canine cancer patients. Hypercoagulability was confirmed as the most common haemostatic abnormality in canine cancer patients and haemostatic dysfunction in canine cancer patients was found related to cancer type and progression of disease. Increase in TEG G, aPTT and fibrinogen were observed in non-mammary carcinomas and were speculated to overall represent a proinflammatory response associated with the disease. Dogs with distant metastatic disease exhibited increased fibrinogen, and d-dimer. Future studies are needed to elucidate the clinical importance of these results.

## Abbreviations

aPTT: activated partial thromboplastin time; AT: antithrombin; CBC: complete blood count; CT scan: × ray computed tomography; DIC: disseminated intravascular coagulation; EDTA: Ethylenediaminetetraacetic acid; FDP: fibrin(ogen) degradation products; NSAIDs: Non-steroidal anti-inflammatory drugs; PT: prothrombin time; PTE: pulmonary thromboembolism; TEG: Thromboelastography; VTE: venous thromboembolism.

## Competing interests

The authors declare that they have no competing interests.

## Authors' contributions

EBA carried out recruitment of patients, data collection and analysis, and drafted the manuscript in association with MT and ATK. BW participated in the data analysis. BM participated in the statistical analysis of the data. ATK conceived of the study and participated in its design. All authors read and approved the final manuscript.

## Supplementary Material

Additional file 1 **The distribution of patients according to cancer type, diagnosis by histopathology and progression of disease**. The table displays the distribution of patients according to cancer type, subdivided by the diagnosis by histopathology, the site of cancer, progression of disease, mean age of the patients, and their sex. Click here for file

Additional file 2 **The distribution of patients according to cancer type, diagnose by histopathology and staging procedures performed**. The table displays the distribution of patients according to cancer type, subdivided by the diagnosis by histopathology and the staging procedures performed. Click here for file

Additional file 3 **Size and site of primary and metastatic cancer for patients with distant metastases**. A list of the included patients with distant metastatic disease describing their site of primary and secondary cancer and paraclinical alteration regarding liver and kidney values evaluated by biochemistry. Click here for file

Additional file 4 **TEG variables, haemostatic and fibrinolytic variables according to cancer type**. Graphic distribution of the TEG values R, K, Angle, Ly30, Ly60, and MA and antithrombin (AT), plasminogen, and haematocrit according to cancer type. Click here for file

Additional file 5 **TEG variables, haemostatic and fibrinolytic variables according to disease progression group**. Graphic distribution of the TEG values R, K, Angle, Ly30, Ly60, and MA and antithrombin (AT), plasminogen, and haematocrit according to disease progression group. Click here for file
