# Supplementary material for: Haemostatic alterations in a group of canine cancer patients are associated with cancer type and disease progression
Source: Acta Vet Scand. 2012 Jan 26;54(1):3. doi: 10.1186/1751-0147-54-3 (PMC3342140; doi:10.1186/1751-0147-54-3)
Supplement: Additional file 2 — The distribution of patients according to cancer type, diagnose by histopathology and staging procedures performed. The table displays the distribution of patients according to cancer type, subdivided by the diagnosis by histopathology and the staging procedures performed. [file 1751-0147-54-3-S2.DOC]

Additional file 2:

The distribution of patients according to cancer type, diagnose by histopathology and staging procedures performed.

| Cancer type | Diagnose by histopathology | N | Anatomic site | Staging procedures performed | | | | | Age*  (range) | Sex† |
| --- | --- | --- | --- | --- | --- | --- | --- | --- | --- | --- |
|  |  |  |  | Autopsy | Thorax X-ray | Thorax CT | Full body CT | Abdominal ultrasound |  |  |
| **Overall** |  | **71** |  |  |  |  |  |  | **8.25**  **(1.5-14.6)** | **F: 52/11**  **M: 19/5** |
| **Carcinoma**  **Mammary** |  | **23** |  |  |  |  |  |  | **8.7**  **(3.5-13.5)** | **F: 23/4** |
|  | Adenocarcinoma | 2 | Mamma |  | 2 |  |  | 1 | 8.4  (7.25-9.5) | F: 2/1 |
|  | Complex carcinoma | 5 | Mamma |  | 5 | 2 |  | 2 | 8.1  (6-9.6) | F: 5/0 |
|  | Tubulopapillary carcinoma | 6 | Mamma | 1 | 6 | 3 |  | 4 | 7.7  (5.3-11.5) | F: 6/1 |
|  | Solid carcinoma | 6 | Mamma |  | 6 | 4 |  | 5 | 8.3  (3.5-12.1) | F: 6/1 |
|  | Adenosquamous carcinoma | 1 | Mamma |  | 1 |  |  |  | 11.4 | F: 1/0 |
|  | Anaplastic carcinoma | 1 | Mamma |  | 1 |  |  | 1 | 12.2 | F: 1/0 |
|  | Carcinoma - unspecified | 2 | Mamma |  | 2 |  |  | 2 | 11.1  (8.8-13.5) | F: 2/1 |
|  |  |  |  |  |  |  |  |  |  |  |
| **Carcinoma**  **Other sites** |  | **7** |  |  |  |  |  |  | **8.9**  **(5.4-11.3)** | **F: 2/1**  **M: 5/1** |
|  | Squamous cell carcinoma | 4 | Cavum nasi |  | 1 | 2 | 1 | 2 | 8.9  (5.4-10.7) | F: 1/0  M: 3/0 |
|  | Anaplastic carcinoma | 1 | Cavum nasi |  |  | 1 |  |  | 11.3 | M: 1/0 |
|  | Poorly differentiated carcinoma | 1 | Subcutis – pharynx region |  |  |  | 1 |  | 6.3 | M: 1/1 |
|  | Carcinoma - unspecified | 1 | Abdomen- Bladder region |  | 1 |  |  | 1 | 9 | F: 1/1 |
|  |  |  |  |  |  |  |  |  |  |  |
| **Osteosarcoma** |  | **6** |  |  |  |  |  |  | **6.7**  **(1.5-12.4)** | **F: 5/1**  **M: 1/0** |
|  | Osteosarcoma | 3 | Bone | 1 | 3 |  |  |  | 7.1  (6.4-8) | F: 3/0 |
|  | Chrondro-blastic osteosarcoma | 1 | Costae | 1 | 1 |  |  |  | 1.5 | M: 1/0 |
|  | Osteosarcoma | 1 | Mamma |  | 1 |  | 1 | 1 | 12.4 | F: 1/0 |
|  | Osteosarcoma | 1 | Maxillaris |  | 1 |  |  | 1 | 5.1 | F: 1/1 |
|  |  |  |  |  |  |  |  |  |  |  |
| **Soft tissue sarcoma** |  | **13** |  |  |  |  |  |  | **9.2**  **(4.6-14.6)** | **F: 9/2**  **M: 4/1** |
|  | Leiomyosarcoma | 1 | Spleen |  | 1 |  |  | 1 | 10.2 | M:1/1 |
|  | Fibrosarcoma | 2 | Cavum nasi |  |  | 2 |  | 1 | 8.5  (6.2-10.8) | M: 2/0 |
|  | Fibrosarcoma | 1 | Mamma |  | 1 |  | 1 |  | 5.5 | F: 1/0 |
|  | Fibrosarcoma | 1 | Skin/subcutis |  |  |  | 1 |  | 7.9 | F: 1/0 |
|  | Peripheral nerve sheat tumor | 1 | Skin/subcutis |  | 1 |  |  |  | 10.5 | F: 1/0 |
|  | Spindle cell sarcoma | 1 | Maxillaris |  | 1 |  |  | 1 | 4.75 | F: 1/0 |
|  | Spindle cell sarcoma | 1 | Skin/subcutis |  | 1 |  | 1 | 1 | 7.3 | F: 1/0 |
|  | Soft tissue sarcoma – unspecified | 4 | Skin/subcutis |  | 4 |  | 2 | 3 | 10.9  (4.6-14.6) | F: 3/1  M: 1/0 |
|  | Soft tissue sarcoma – unspecified | 1 | Mandible |  |  |  | 1 |  | 12.75 | F: 1/1 |
|  |  |  |  |  |  |  |  |  |  |  |
| **Masto-cytoma** |  | **12** |  |  |  |  |  |  | **7.6**  **(1.7-12.3)** | **F: 7/1**  **M: 5/2** |
|  | Mastocytoma grade 2 | 4 | Skin/subcutis |  | 3 |  |  | 4 | 7.6  (4.4-10) | F: 3/0  M: 1/0 |
|  | Mastocytoma grade 3 | 2 | Skin/subcutis |  |  |  |  | 2 | 5.4  (1.7-9.3) | F: 2/0 |
|  | Mastocytoma –  Diagnosed by cytology | 6 | Skin/subcutis |  | 4 |  |  | 6 | 8.3  (3.1-12.3) | F: 2/1  M: 4/2 |
|  |  |  |  |  |  |  |  |  |  |  |
| **Lymphoma** |  | **10** |  |  |  |  |  |  | **7.3**  **(4.2-12.4)** | **F: 6/2**  **M: 4/1** |
|  | Lymphoblastic   - B cell origin - Multicentic | 4 | Stage IIIb: 1  Stage IVb: 2  Stage Vb: 1 |  | 2  1 |  | 1 | 1  2  1 | 6.9  (4.2-12.4) | F: 3/0  M: 1/0 |
|  | Lymphoblastic   - T cell origin - Multicentric | 1 | Stage IIIb: 1 |  | 1 |  |  | 1 | 5.6 | M: 1/0 |
|  | Lymphoblastic   - origin unknown - Multicentic | 3 | Stage IIb: 1  Stage IIIb: 2 |  | 2 |  |  | 1  2 | 6.5  (5.9-7.4) | F: 2/2  M: 1/1 |
|  | Lymphoblastic   - origin unknown - Mediastinal | 1 | Stage Ib: 1 |  | 1 |  |  | 1 | 8.4 | F: 1/0 |
|  | Lymphocytic   - unknown origin - Alimentary | 1 | Stage: IVb: 1 |  | 1 |  |  | 1 | 11.6 | M: 1/0 |

* Mean age is given in years with range in brackets.

† Number of patients of each sex. F=female, M=male. Numbers following / is number of neutered patients.
