# Supplementary material for: Haemostatic alterations in a group of canine cancer patients are associated with cancer type and disease progression
Source: Acta Vet Scand. 2012 Jan 26;54(1):3. doi: 10.1186/1751-0147-54-3 (PMC3342140; doi:10.1186/1751-0147-54-3)
Supplement: Additional file 3 — Size and site of primary and metastatic cancer for patients with distant metastases. A list of the included patients with distant metastatic disease describing their site of primary and secondary cancer and paraclinical alteration regarding liver and kidney values evaluated by biochemistry. [file 1751-0147-54-3-S3.DOC]

Additional file 3:

Size and site of primary and metastatic cancer for patients with distant metastases.

| **Primary tumor/**  **Cancer type** | **Diagnose by histopathology** | **Anatomic site** | **Site of distant metastases** | **Size (max) of distant metastases** | **Liver affection**  **(biochemistry)** | **Azotemia (biochemistry)** |
| --- | --- | --- | --- | --- | --- | --- |
| Carcinoma  Mammary | Tubulopapillary carcinoma | Mamma | Spleen | Ø 1 cm | No | No |
| Carcinoma  Mammary | Solid carcinoma | Mamma | Lungs | Ø 0.5 cm | No | No |
| Carcinoma  Mammary | Solid  Carcinoma | Mamma | Sternal lymph nodes | Ø 3 cm | No | No |
| Carcinoma  Mammary | Carcinoma – unspecified | Mamma | Abdominal lymph nodes | Ø 3 cm | No | No |
| Carcinoma  Other | Anaplastic carcinoma | Cavum nasi | Lungs | Ø 6 cm | No | No |
| Carcinoma  Other | Squamous cell carcinoma | Cavum nasi | Lungs | Ø 1 cm | No | No |
| Carcinoma  Other | Carcinoma  Unspecified | Abdomen -  Bladder  region | Liver,  Spleen,  Abdominal lymph nodes | Ø 2 cm | Yes | No |
| Osteosarcoma | Osteosarcoma | Proximal Tibia | Lungs | Ø 3 cm | No | No |
| Osteosarcoma | Chrondro-blastic osteosarcoma | Costae | Lungs | Ø 1 cm | No | No |
| Osteosarcoma | Osteosarcoma | Maxillaris | Lungs  Liver | Ø 4 cm | Yes | No |
