# Supplementary material for: Haemostatic alterations in a group of canine cancer patients are associated with cancer type and disease progression
Source: Acta Vet Scand. 2012 Jan 26;54(1):3. doi: 10.1186/1751-0147-54-3 (PMC3342140; doi:10.1186/1751-0147-54-3)
Supplement: Additional file 5 — TEG variables, haemostatic and fibrinolytic variables according to disease progression group. Graphic distribution of the TEG values R, K, Angle, Ly30, Ly60, and MA and antithrombin (AT), plasminogen, and haematocrit according to disease progression group. [file 1751-0147-54-3-S5.DOC]

Additional file 5:

**TEG variables, haemostatic and fibrinolytic variables according to disease progression group**
